# Practices of Trans-National Corporations: The Need to Change Global Economic and Political Norms: Comment on "National Public Health Surveillance of Corporations in Key Unhealthy Commodity Industries – A Scoping Review and Framework Synthesis"

**DOI:** 10.34172/ijhpm.8660

**Published:** 2024-09-09

**Authors:** Fran Baum, Julia Anaf

**Affiliations:** Stretton Health Equity, Stretton Institute, University of Adelaide, Adelaide SA, Australia.

**Keywords:** Commercial Determinants, Governance, Norms, Power, Advocacy, Political Economy of Health

## Abstract

Trans-national corporations (TNCs) are recognised as having an adverse impact on public health through the marketing and sale of unhealthy products. In addition to this some of their practices affect health, including taxation avoidance, lobbying politicians to gain favourable legislative and regulatory environments for their operations, and failure to abide by occupational health and safety standards. We argue that while considering the individual practices of commercial actors is crucial the true public health harms are only evident when the synergistic impacts of the practices are considered. We also note that the practices are supported by a global economic and political system which operates in the favour of commercial actors rather than the health of people and the planet. Consequently there needs to be a norm change by which norms and power are shifted away from commercial interests and externalities of commercial practices are no longer outsourced to the public purse.

## Introduction

Commercial determinants of health are coming under increasing attention in public health academic literature and practice. The World Health Organization (WHO) now has a unit devoted to their consideration and increasing concern is being raised about the adverse impact of trans-national corporations (TNCs) on health. Bennett and colleagues’^1^ paper provides a helpful review on national public health surveillance of unhealthy commodity industries (UCI). Their deductive synthesis used Madureira Lima and Galea’s “vehicles of power”^2^ as a framing to identify fourteen frameworks designed to identify or monitor how corporate practices influenced health outcomes in relation to ultra processed food, tobacco, and alcohol. Their research identified 37 corporate practices grouped under five main headings whereby they influence crucial environments: political (eg, lobbying and tax avoidance); preference shaping (eg, corporate social responsibility; civil society capture); influencing the knowledge environment (eg, funding research); legal (pre-emptive litigation) extra-legal (eg, harassment). What is striking about these five sets of practices is that they would be relevant to many other corporate players including the fossil fuel industry which uses all these practices to persist in mining products contributing to global warming and the pharmaceutical industry which uses many of these practices to protect their intellectual property despite much public funding supporting the discovery and public health emergencies. The practices of these TNCs mirror those of UCI raising questions about the extensive power of all TNCs.

 Gilmore et al^3^ pinpoint two further types of practices which are used by TNCs (including the UCI) in addition to those identified by Bennett et al. They are adverse labour and employment practices and supply chain and waste practices. In regard to the UCI an example of poor employment practices is seen in fast food restaurants in the United States where employees are on very low wages.^4^ Supply chain issues are evident in the food production chains where exploitative employment practices are hidden upstream (for example for small scale farmers) and TNCs can distance themselves from these.^5^ While detailing these practices is very helpful for public health advocates seeking to create healthy food systems as it reveals the extent to which corporate actors go to in order to impede such a system, it does not capture the extent to which TNCs are dominating the global economic system. There is synergistic effect between the practices which amplifies their health impact. This domination is a major threat to public health and we examine its nature in the following section.

###  Do Corporations Rule the World?

In 1995 David Korten^6^ published a foresightful book entitled *“When Corporations Rule the World”* where he predicted that the power of these entities would increase and they would become like feudal overlords yielding similar autocratic power. The practices identified by Bennett et al are an important part of a global economic environment which is very favourable to the operations and profit taking of TNCs. But they are only part of the picture, as Gilmore et al show in their discussion of how the global economic and political architecture promotes the interest of the TNCs. Their analysis highlights the importance of underlying drivers in this global architecture which promotes the interest of the corporate sector including UCIs. These are: norms that are shaped in the interests of the commercial elites, the relative weakening of the power of nation states compared to the commercial sector, and how corporations externalise some of their costs to nation states and society. It is these norms which enable UCIs to undertake their health harming practice. Taken together these underlying drivers do indicate that Korton’s prediction is coming true! We briefly examine each of these norms in relation to the UCI.

The weakening of the power of nation states means governance of the practices of UCIs is limited. Forty years of neo-liberal public policies has meant that public sectors have been reduced in size and the capacity for regulation greatly reduced.^7^ The UCI practices themselves (especially lobbying) have been responsible for the implementation of neo-liberal public policies and the creation of the norm that small government is desirable.

## Norms and the Commercial Elite

Hand in glove with the dilution of the power of nation states has been the shaping of norms in the interests of the commercial elite. A powerful set of actors is making this happen through the operation of the global consulting firms. These firms are both a product and outcome of neoliberal polices, with commercial actors benefitting from the roles and services provided by these entities. In distinction to TNCs, they are unincorporated private partnerships which offer a wide range of services including auditing and taxation advice to private entities and to governments. Most of the services offered by consultancy firms are provided by the Big Four’ accounting/professional services firms (PwC, Deloitte, EY, KPMG) and the ‘Big Three’ management and strategy firms (McKinsey, Boston Consulting, Bain and Co). They have been assessed as having a significant adverse impacts on society including on health.^8^ Negative impacts arise from the conflicts of interest in offering financial advice as well as auditing services. Other negative aspects include the growing influence of these companies over government policy by promoting neoliberal values including privatisation and outsourcing of public assets. This undermines the power of the state and ability of public servants to enact policies in the public good. Their role in undermining the capacity of public services has been noted^8^ including through an Australian Parliamentary Committee^9^ where submissions have detailed this impact. Consulting companies also play a key role in advising UCIs how to reduce their taxation liabilities, so reducing the funds available for public goods in many countries. The opportunities for conflicts of interest are multiple. Low-income workers may be negatively affected by global consulting firms which advise TNCs on cost-cutting through downsizing, outsourcing, mergers and acquisitions, or restructuring, which can lead to growing inequality and poverty. Consultancies can also reframe issues for UCIs in a way that has been described as “polishing facts.” This is seen in relation to sustainability reporting where they are tasked with ensuring a company “ticks the box” and fulfils its reporting requirements, yet often the information presented can be superficial and lack context.^10^

The final norm we consider is that of externalising corporate costs to governments and society in general. In the case of UCIs examples include excessive litter from fast food outlets and their substantial contribution to the accelerating non-communicable disease burden. The costs arising are incurred by governments which in healthcare are massive. Further examples are the ecological devastation of agribusiness which underpins the commodities used in the production of ultra-processed foods.^5^ This form of agriculture has undermined small farmers and also affected multiple eco-systems.

## New Norms – Effective Governance

 Our consideration of the norms that allow UCIs to continue their health harming practices indicate that if these are to be effectively governed then a shift in the norms of the global economic and political system described by Gilmore et al^3^ is required (see Figure). Political science recognises that norm shifts can happen and can happen swiftly.^11^

**Figure:**
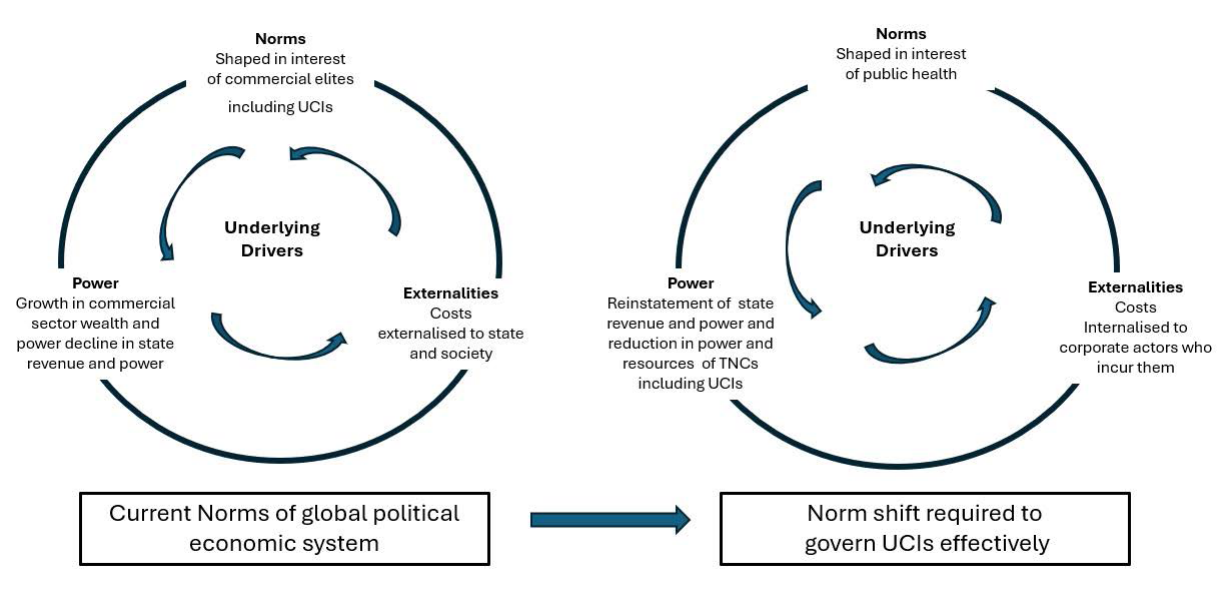
Norms of Global Political Economic System: Current and potential. Abbreviation: UCIs, unhealthy commodity industries. Source: Gilmore et al.^3‎^

 In finishing this commentary we note that strong action is required to counter the norms underlying commercial domination of the global political economy. Nation states have been largely captured by the commercial, and especially corporate agenda, and are unlikely to garner the political will for a norm cascade unless there is sufficient external pressure on them. The voices that do call out the growing power and influence of the corporate sector are from progressive civil society.

 Thus, investigative journalists, trade unions, and civil society advocacy groups use a range of tactics which can include naming and shaming TNCs who are engaged in health harming practices. An example is the ways in which local civil society activists lobbied against the establishment of a McDonalds in their local community.^4^ In instances where TNCs engage in illegal behaviour, investigative journalists such as Michael West^12^ examine multiple examples of corporate malfeasance. Global civil society organisations also make open strident critiques of TNC behaviour. A recent example is the People’s Health Movement Call to Action from the 5^th^ People’s Health Assembly (PHA5 Mar del Plata 2024 Call to Action) which has a section on “A world free of corporate control: Resisting corporatisation, marketisation, financialisation and colonisation.” An example of the content is “Governments are colluding with and appear subservient to, the power of these entities (TNCs) and provide wide-ranging forms of corporate welfare including bailouts, subsidies, and reduced taxation.” A further example is the Global Health Watch a project led by the People’s Health Movement which in its 6^th^ Edition had a section on “Confronting the Commercial Determinants.”

 Such actors who question the operation of 21st century capitalism which underpins and supports the activities of TNCs^13^ will be vital to establishing a global political economy with norms which work in the interest of health rather than profits. Establishing healthy food systems requires a world in which corporate interests are not underwritten by norms which legitimise the practices Bennett et al identify.

## Ethical issues

 Not applicable.

## Conflicts of interest

 Authors declare that they have no conflicts of interest.
